# Editorial: Cross-kingdom communications among plants, fungi, and bacteria: from molecules to ecological factors

**DOI:** 10.3389/fpls.2026.1862454

**Published:** 2026-05-11

**Authors:** Xianan Xie, Feiyun Xu, Luisa Lanfranco, Jia-Jia Han, Kai Sun

**Affiliations:** 1 Jiangsu Key Laboratory for Pathogens and Ecosystems, Jiangsu Engineering and Technology Research Center for Industrialization of Microbial Resources, College of Life Sciences, Nanjing Normal University, Nanjing, China; 2 State Key Laboratory of Conservation and Utilization of Subtropical Agro-Bioresources, Guangdong Key Laboratory for Innovative Development and Utilization of Forest Plant Germplasm, College of Forestry and Landscape Architecture, South China Agricultural University, Guangzhou, China; 3 Center for Plant Water-use and Nutrition Regulation and College of Resources and Environment, Joint International Research Laboratory of Water and Nutrient in Crop, Fujian Agriculture and Forestry University, Fuzhou, China; 4 Department of Life Sciences and Systems Biology, University of Turin, Turin, Italy; 5 State Key Laboratory of Vegetation Structure, Function and Construction (VegLab); Ministry of Education Key Laboratory for Transboundary Ecosecurity of Southwest China; Yunnan Key Laboratory of Biological Adaptation, Conservation and Utilization, Yunnan University, Kunming, China

**Keywords:** cross-kingdom effectors, mycorrhizal fungi, nutrient exchange, plant physiology and metabolites, plant-fungi-bacteria interactions, rhizobacteria

In modern agriculture and forestry, the imperative for sustainable development demands innovative strategies that reduce chemical inputs while maintaining productivity. Central to this challenge is a deeper understanding of the complex, multi-trophic interactions occurring within the rhizosphere and plant tissues (Ling et al., 2022). The associations between plants and beneficial microbes particularly mycorrhizal fungi (**Figure 1**), and plant growth-promoting rhizobacteria, are well-recognized for their profound impacts on nutrient acquisition, stress tolerance, and overall ecosystem functioning (Trivedi et al., 2020; Ma et al., 2026). More recently, the conceptual framework has expanded to recognize the plant-arbuscular mycorrhizal (AM) fungus-bacterium continuum, wherein AM fungi-associated endobacteria or hyphosphere-associated bacteria actively modulate the outcome of plant-fungal symbioses (Duan et al., 2024). However, the molecular dialogues and ecological principles governing this cross-kingdom communication remain in their infancy (Silvestri et al., 2025; Usländer et al., 2026).

**Figure 1:**
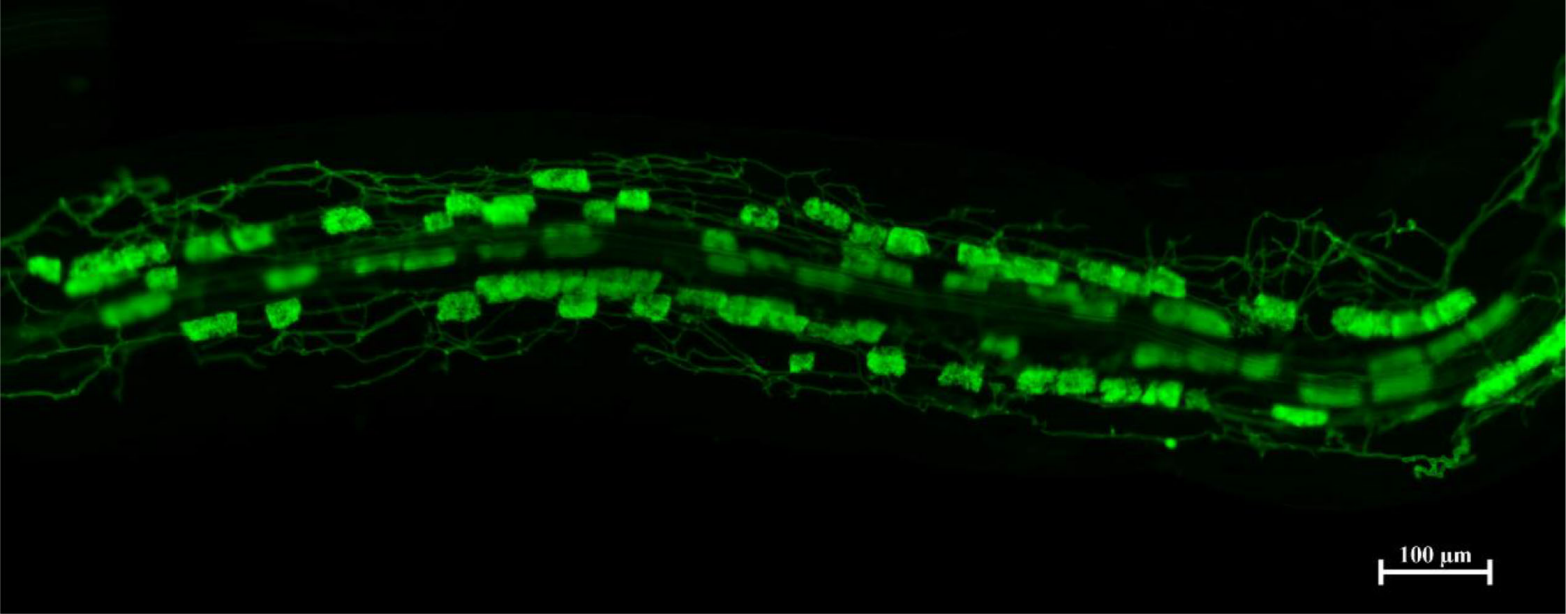
WGA488 staining of a *Medicago truncatula* root colonized by the mycorrhizal fungus *Rhizophagus irregularis* showing the presence of arbuscules in the cortical parenchima.

This Research Topic, “Cross-Kingdom Communications Among Plants, Fungi and Bacteria: From Molecules to Ecological Factors”, brings together a compelling collection of 14 original research articles that collectively advance our understanding of how plants both shape and are shaped by their associated microbiomes across diverse environments, from deserts to subtropical forests and agricultural fields. The contributions herein underscore the intricate signaling networks, environmental drivers, and functional consequences of these interactions, while highlighting promising translational applications for sustainable agriculture and ecosystem conservation.

## Signaling molecules and chemotaxis in the rhizosphere

The ability to locate and migrate toward plant roots is a fundamental prerequisite for microbes to establish either beneficial or pathogenic associations. Keren et al. demonstrate that root-secreted nucleosides act as potent chemoattractants for a diverse range of rhizobacteria, including both plant growth-promoting *Bacillus* strains and the pathogen *Xanthomonas campestris*. This work expands the repertoire of known rhizosphere signaling molecules beyond conventional nutrients and suggests that purinergic signaling may represent a conserved, cross-kingdom communication mechanism that shapes the assembly of the root microbiome.

## Environmental and spatial drivers of microbial community assembly

A recurring theme across this Research Topic is the critical importance of environmental and spatial factors in structuring plant-associated microbial communities. Several studies leverage high-throughput sequencing to disentangle these complex relationships. Zhao et al. reveal that spatial variation, driven by geographic distance and contrasting soil properties such as pH and total nitrogen (N), exerts a stronger influence than seasonality on the assembly of fungal communities in *Larix gmelinii* forests. This spatial heterogeneity dictates the balance between deterministic and stochastic processes, with implications for forest health management.

This principle extends to the rhizocompartments of diverse plant species. Mo et al. demonstrate that the rhizocompartment (endosphere, rhizosphere, bulk soil) primarily drives fungal community structure across different life forms of halophytes in an arid wetland. They observe a clear niche differentiation, with endophytic compartments enriched for salt-tolerant genera such as *Aporospora* and *Monosporascus*, highlighting how plants filter and select specific fungal taxa from the surrounding soil matrix. Similarly, Xie et al. provide a detailed characterization of the microbiome associated with the medicinal orchid *Dendrobium huoshanense*, showing a marked decline in fungal diversity from the cultivation medium to the root endosphere. This gradient reflects the strong selective pressure exerted by the host plant (Liu et al., 2021), favoring specialist endophytic fungi while largely excluding saprotrophs and potential pathogens. The predominance of functionally uncharacterized fungi within the endosphere underscores a vast, unexplored reserve of biological diversity with potential implications for the orchid’s medicinal properties. Extending this theme, Tang et al. investigate the rhizosphere microbiomes of five *Impatiens* species across an elevational gradient. Their findings highlight pronounced interspecific variation, with endemic species harboring distinct fungal communities strongly correlated with elevation and soil nutrients.

The importance of geographic origin is further exemplified in the studies of Taheri et al. and Chen et al. Taheri et al. characterize the culturable endophytic fungal diversity of hairy vetch (*Vicia villosa*) across Japan, revealing that soil pH and geographic location significantly shape community composition. Their functional screening identifies numerous isolates with plant growth-promoting traits, including a novel strain of *Penicillium griseofulvum* with potential as a bioinoculant. Chen et al. link the rhizosphere bacterial community of the medicinal plant *Zanthoxylum nitidum* to its geographic origin, and, critically, establish a positive correlation between the abundance of specific bacterial genera (e.g., *Rudaea*, *Bradyrhizobium*) and the accumulation of the key bioactive compound, nitidine chloride. This study provides a tangible link between soil microbiota and the quality of medicinal plant materials.

Extending beyond the rhizosphere of annual crops, Quan et al. provide a compelling case study of plant-soil feedback in an extreme desert environment. Focusing on the endangered relict shrub *Tetraena mongolica*, a keystone species in the West Ordos region of China, the authors demonstrate that the plantation of *T. mongolica* profoundly ameliorates desert soil properties. These physicochemical improvements were paralleled by marked shifts in the soil microbiome, with increased bacterial and fungal diversity indices in the root-zone soil. Notably, this study revealed that *T. mongolica* simplified bacterial co-occurrence networks while increasing the complexity of fungal networks, suggesting a differential restructuring of microbial interactions. This work highlights the irreplaceable role of keystone relict plants in desert ecosystem stability and reveals how ancient plant lineages engineer their soil environment for long-term persistence.

## Functional mechanisms of symbiosis: from nutrient exchange to disease resistance

The functional core of plant-microbe interactions lies in the exchange of resources and the modulation of host physiology (Trivedi et al., 2020). Zadegan et al. employ comparative transcriptomics to dissect the differential compatibility between *Bradyrhizobium* strains and soybean (cultivated versus wild). This study reveals that the formation of non-functional nodules is associated with the activation of defense-related and cell wall biogenesis genes, offering a molecular blueprint for understanding symbiotic specificity and domestication effects.

Mycorrhizal symbiosis is a central pillar of nutrient acquisition in terrestrial ecosystems (Duan et al., 2024). Li et al. provide a compelling demonstration of how arbuscular mycorrhizal (AM) fungi modulate the N nutrition of *Eucalyptus* seedlings. They show that mycorrhization shifts the plant preference from complex organic N sources (e.g., peptone) to simpler forms (e.g., glycine), and this is accompanied by the upregulation of specific organic N transporters (e.g., EgAAP3, EgLHT1). This work reveals a sophisticated level of metabolic coordination that enhances N use efficiency under low-fertilization regimes.

Beyond nutrition, beneficial microbes can prime plant defenses. Zhang et al. characterize the W-box binding (WRKY) transcription factor family in *Eucalyptus grandis* and demonstrate that colonization by the AM fungus *Rhizophagus irregularis* induces the expression of specific *EgWRKY* genes. This transcriptional reprogramming is associated with enhanced defense against the bacterial pathogen *Ralstonia solanacearum*, illustrating the role of AM symbiosis in bolstering the plant’s innate immune system.

The specificity of symbiotic dependency is elegantly contrasted in the work of Yang et al. on two medicinal orchids. *Pleione bulbocodioides* strictly depends on mycorrhizal fungi for complete development from germination to seedling, whereas *Bletilla striata* can germinate autonomously, with fungi greatly accelerating the process. This dichotomy highlights the evolutionary spectrum of symbiotic reliance and provides practical insights for the conservation and cultivation of these valuable species.

## Translational applications for sustainable agriculture

The ultimate goal of understanding these fundamental plant-microbe interactions is to harness them for practical applications. Two studies in this Research Topic showcase innovative, technology-driven approaches to improving agricultural sustainability. Liu et al. demonstrate that optimizing nitrate supply can enhance rice resistance to bacterial leaf blight. Through transcriptomic analysis, they elucidate a molecular regulatory network wherein nitrate modulates defense gene expression, providing a rational basis for nutrient management strategies that reduce reliance on chemical pesticides.

Furthermore, Bian et al. (2025) explore a novel irrigation technique using micro-/nanobubble oxygenation. They show that this method significantly enhances soil phosphorus availability and maize yield by altering the structure and diversity of the rhizosphere bacterial community and increasing alkaline phosphatase activity. This study exemplifies how manipulating the soil physicochemical environment can indirectly steer the microbiome to improve nutrient cycling and crop productivity.

In conclusion, this Research Topic significantly advance our understanding of the intricate cross-kingdom communications among the plants, fungi, and bacteria. They illuminate the signaling molecules that initiate these interactions, the environmental and host genetic factors that sculpt microbial communities, and the functional mechanisms by which these associations enhance nutrient uptake and resilience to stress. As we move forward, integrating this knowledge into ecological models and agricultural practices will be essential for developing robust, microbiome-informed strategies that promote both ecosystem health and sustainable food and fiber production.

As we are writing this Editorial, less than 2 years after the acceptance of the first manuscript, this Research Topic has gained over 42,000 views and 9,500 downloads. This success mirrors a growing and widespread recognition within the scientific community of the fundamental importance of cross-kingdom communications in shaping plant health and ecosystem resilience. The enthusiastic reception of this Research Topic underscores the urgent need to move beyond single-organism perspectives and embrace the complexity of the plant symbiosis, as we collectively strive to translate these intricate ecological insights into practical solutions for a more sustainable agricultural and environmental future.

## References

[B1] Bian Q. Dong Z. Zhao Y. Feng Y. Fu Y. Wang Z. . (2025). Micro-/nanobubble oxygenation irrigation enhances soil phosphorus availability and yield by altering soil bacterial community abundance and core microbial populations. Front. Plant Sci. 15, 1497952. doi:10.3389/fpls.2024.1497952, PMID: 40007768 PMC11851534

[B2] Duan S. Feng G. Limpens E. Bonfante P. Xie X. Zhang L. (2024). Cross-kingdom nutrient exchange in the plant-arbuscular mycorrhizal fungus-bacterium continuum. Nat. Rev. Microbiol. 22, 773–790. doi:10.1038/s41579-024-01073-7, PMID: 39014094

[B3] Ling N. Wang T. Kuzyakov Y. (2022). Rhizosphere bacteriome structure and functions. Nat. Commun. 13, 836. doi:10.1038/s41467-022-28448-9, PMID: 35149704 PMC8837802

[B4] Liu X. Jia P. Cadotte M. W. Zhu C. Si X. Wang Y. . (2021). Host plant environmental filtering drives foliar fungal community assembly in symptomatic leaves. Oecologia 195, 737–749. doi:10.1007/s00442-021-04849-3, PMID: 33582871

[B5] Ma Y. Li X. Luo Y. M. (2026). Microbiome-driven innovations for climate-resilient crop production. Nat. Food. 7, 304–308. doi:10.1038/s43016-026-01337-w, PMID: 41933264

[B6] Silvestri A. Ledford W. C. Fiorilli V. Votta C. Scerna A. Tucconi J. . (2025). A fungal sRNA silences a host plant transcription factor to promote arbuscular mycorrhizal symbiosis. New Phytol. 246, 924–935. doi:10.1111/nph.20273, PMID: 39555692 PMC11982788

[B7] Trivedi P. Leach J. E. Tringe S. G. Sa T. Singh B. K. (2020). Plant–microbiome interactions: from community assembly to plant health. Nat. Rev. Microbiol. 18, 607–621. doi:10.1038/s41579-020-0412-1, PMID: 32788714

[B8] Usländer A. Haag M. V. Cheng A. P. Lederer B. Khoo J. Y. Dunker F. . (2026). Cross-kingdom RNA interference promotes arbuscular mycorrhiza development. Nat. Plants. 12, 695–702. doi:10.1038/s41477-026-02247-2, PMID: 41813826 PMC13106029

